# MOViDA: multiomics visible drug activity prediction with a biologically informed neural network model

**DOI:** 10.1093/bioinformatics/btad432

**Published:** 2023-07-11

**Authors:** Luigi Ferraro, Giovanni Scala, Luigi Cerulo, Emanuele Carosati, Michele Ceccarelli

**Affiliations:** Sylvester Comprehensive Cancer Center, University of Miami, Miami, FL 33131, United States; BIOGEM Institute of Molecular Biology and Genetics, 83031 Ariano Irpino, Italy; Department of Biology, University of Naples “Federico II”, 80128 Naples, Italy; BIOGEM Institute of Molecular Biology and Genetics, 83031 Ariano Irpino, Italy; Department of Science and Technology, University of Sannio, 82100 Benevento, Italy; Department of Chemical and Pharmaceutical Sciences, University of Trieste, 34127 Trieste, Italy; Sylvester Comprehensive Cancer Center, University of Miami, Miami, FL 33131, United States; Department of Public Health Sciences, Miami, FL 33131, United States

## Abstract

**Motivation:**

The process of drug development is inherently complex, marked by extended intervals from the inception of a pharmaceutical agent to its eventual launch in the market. Additionally, each phase in this process is associated with a significant failure rate, amplifying the inherent challenges of this task. Computational virtual screening powered by machine learning algorithms has emerged as a promising approach for predicting therapeutic efficacy. However, the complex relationships between the features learned by these algorithms can be challenging to decipher.

**Results:**

We have engineered an artificial neural network model designed specifically for predicting drug sensitivity. This model utilizes a biologically informed visible neural network, thereby enhancing its interpretability. The trained model allows for an in-depth exploration of the biological pathways integral to prediction and the chemical attributes of drugs that impact sensitivity. Our model harnesses multiomics data derived from a different tumor tissue sources, as well as molecular descriptors that encapsulate the properties of drugs. We extended the model to predict drug synergy, resulting in favorable outcomes while retaining interpretability. Given the imbalanced nature of publicly available drug screening datasets, our model demonstrated superior performance to state-of-the-art visible machine learning algorithms.

**Availability and implementation:**

MOViDA is implemented in Python using PyTorch library and freely available for download at https://github.com/Luigi-Ferraro/MOViDA. Training data, RIS score and drug features are archived on Zenodo https://doi.org/10.5281/zenodo.8180380.

## 1 Introduction

Large-scale genomic studies have been instrumental in understanding recurrent somatic genetic alterations within cancer cells and for the characterization of their functional effects in transformed cells (Sanchez-Vega *et al.* 2018). One of the main challenges consists into exploiting this molecular characterization to identify therapeutic targets and develop personalized therapies (Dezső and Ceccarelli 2020). Machine learning (ML) models can exploit multimodal screening datasets to develop predictive algorithms that associate omics features with responses (Garnett *et al.* 2012). There have been several attempts to utilize the data from these screenings in various ML frameworks, such as Variational Autoencoders (Rampášek *et al.* 2019), Deep Networks (Preuer *et al.* 2018, Chiu *et al.* 2019), Convolutional Neural Networks (Cortés-Ciriano and Bender 2019, Liu *et al.* 2019), ensemble Neural Network models (Tan *et al.* 2019), and a combination of these approaches with different encodings of the features (Menden *et al.* 2019) to predict the half-maximal inhibitory concentration (IC_50_) (Baptista *et al.* 2021). In general, the drug sensitivity prediction models can be classified into single-drug learning and multidrug learning (Firoozbakht *et al.* 2022). The latter are particularly challenging since drugs accounted for greater part of the variance in drug response values, while cell lines accounted for only a small proportion of variability (Shen *et al.* 2023). Multidrug learning models typically contain three components: cell line embedding, to encode molecular profiles such as gene expression, mutation status, and copy number variation; drug embedding, used to encode drug representation features such as string, fingerprint, or graph (An *et al.* 2022); and finally a drug sensitivity prediction module that uses cell and drug embeddings to estimate the effect in terms of IC_50_ and/or the area under the dose–response curve (AUC). Most of these studies used ML models as “black boxes” optimized for prediction accuracy without the possibility of interpreting the biological mechanisms underlying predicted outcomes. However, one commonly needs to understand the rules behind model predictions, mainly when the final goal is to prioritize drugs (or drug combinations) for use in clinical trials. Recently, Ideker et al. proposed a “visible neural network” (VNN) to address this issue (Kuenzi *et al.* 2020). The model, called *DrugCell*, encodes cell genotypes into a trainable network composed of modules organized according to the Biological Process Gene Ontology (GO) hierarchy, where each module is associated with a specific GO term and connected to the nodes (genes) annotated with that specific term. Interpreting the activity of each module allows the association between specific biological pathways and drug response to be discovered. DrugCell was one of the first attempts to use *interpretable ML* for drug sensitivity prediction. Nevertheless, there are several possibilities to extend and improve this biologically informed approach. First, DrugCell relies on the somatic single-nucleotide variation profiles of the screened models. Second, it is vital to consider the imbalanced nature of the data since, in almost all available large-scale screening repositories, results sensitivity assays tend to be skewed toward values representing lack of sensitivity, with a small minority representing the sensitivity of a cell line to specific drugs. Classical ML algorithms typically assume a balanced class distribution or equal misclassification costs, which is rarely the case in real-world scenarios. Different learning strategies, including cost-sensitive learning, sampling methods, and ensemble learning have been proposed to deal with the imbalanced data including the significance of evaluation metrics used for imbalanced learning (Haixiang *et al.* 2017). Here, we propose an approach to drug activity prediction using a Multi-Omics Visible Drug Activity prediction model, or MOViDA that extends the existing DrugCell’s visible network approach by incorporating pathway activity from gene expression and copy number variation data. This allows a more comprehensive characterization of the biological models, leading to more accurate predictions of drug activity. The training algorithm accounts for the skewness in the input dataset with a random sampler based on a multinomial distribution. Our approach falls within the random oversampling and undersampling class of methods to learning from imbalanced data (He and Garcia 2009). Moreover, MOViDA enhances the interpretability of drug descriptions using fingerprints and molecular descriptors. These descriptors relate the 3D molecular structures of drugs to their physicochemical and pharmacokinetic properties, making it easier to understand the impact of a drug on a biological system. The results of our study show that MOViDA outperforms the existing models in predicting drug sensitivity, particularly for favorable treatments. To further enhance the biological interpretability of the model, we developed an *ad hoc* network explanation method to score the pathways affecting sensitivity predictions in specific sets of cell lines. Finally, we extended MOViDA to perform prediction of drug synergy.

## 2 Materials and methods

### 2.1 Network architecture

**Figure 1.**
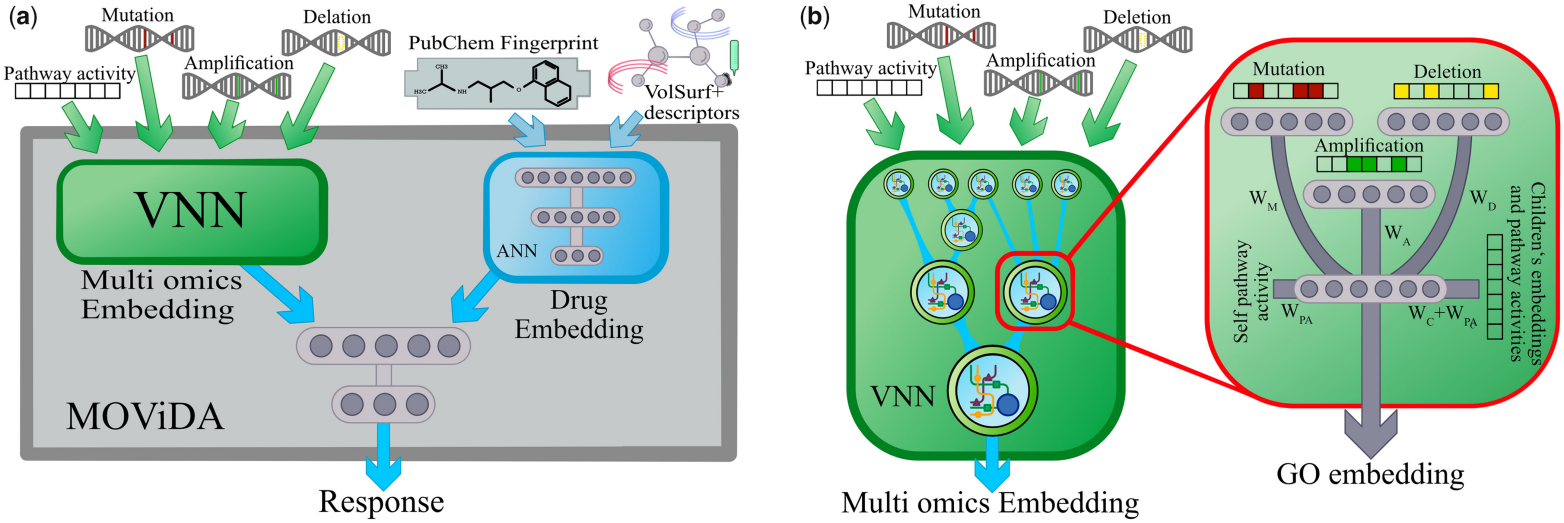
MOViDA architecture. (a) The network is composed of three distinct subnetworks. The multiomics embedding net takes in input multiomics profiles of a cell line model. In contrast, the drug embedding net receives the drug description, composed of PubChem fingerprints and VolSurf+ molecular descriptors. The final layers combine the embeddings and predict the AUC. (b) The Multiomics Embedding net comprises a set of modules, each representing a specific Gene Ontology term. The modules are connected according to the Biological Process Gene Ontology Hierarchy. Each GO sub-module takes in input the multiomics profile of a cell line model, considering only the genes associated to former

MOViDA is a feedforward deep neural network that predicts the drug sensitivity of a cell line. Different omics assays represent each cell line. The structure of the whole Neural Network is separated into two branches (Fig. 1a): a *VNN* and a feedforward artificial neural network (*ANN*). The *ANN* on the right branch is a neural network taking as input a combination of PubChem fingerprints and molecular descriptors relating 3D molecular shape with physical–chemical and pharmacokinetic properties (Crivori *et al.* 2000). Drug features are encoded into a three-layer neural architecture with 100, 50, and 6 nodes. The *VNN* (Fig. 1b) on the left branch represents the Biological Process hierarchy of the GO composed of five layers, one element as a root, and a total of 2086 GO terms. Each GO term is connected to more generic GO ancestors (at least one) and is represented by a sub-submodule composed of a set of *k *+* *1 nonlinear units. *k* units are connected to the input layer and the output of previous layers, here we use the same value of *k *=* *6 as in DrugCell. Each unit also receives a normalized gene set enrichment score (NES) of that GO term computed on gene expression, this value is concatenated with the activation of the *k* units and fed to the next layer in the hierarchy. The input layer is composed of nodes of three different kinds: mutations, amplification, and deletions. Each GO submodule is connected to the input genes annotated with that term.

The activation of the units at the root of the hierarchy represents a multiomic embedding of the cell line. The training phase aims at learning the weights of each subsystem. In particular, every unit of each module *s* has the following output: where *M_s_*, *A_s_*, and *D_s_* are the binary vectors that describe the mutation, amplification, and deletion status of the genes associated with the subsystem *s* and *W_M_*, *W_A_*, and *W_D_* are the corresponding weights; *W*_NES_ is the weight of the normalized enrichment score NES_*s*_ of the term *s* resulting from gene expression; *W_c_* and $W_{{\text{NES}}_{c}}$ are the weights associated to the embedding *E_c_* and NES_*c*_ of child *c* of the considered subsystem. *E_s_* is the embedding of a subsystem *s*, which is a nonlinear transformation *f* of the inputs consisting of hyperbolic tangent and batch normalization, and *b_s_* is the bias term.


(1) $$\begin{array}{c} {\text{IM}}_{s}=W_{M}M_{s}{\text{IA}}_{s}=W_{A}A_{s}{\text{ID}}_{s}=W_{D}D_{s}\\ INES_{s}=W_{\text{NES}}{\text{NES}}_{s}\;IC_{c}=W_{c}E_{c}+W_{{\text{NES}}_{c}}{\text{NES}}_{c}\\ E_{s}=f({\text{IM}}_{s}+{\text{IA}}_{s}+{\text{ID}}_{s}+\mathrm{INE}S_{s}+\sum_{c\in \mathrm{desc}(s)}IC_{c}+b_{s}) \end{array},$$

A third neural network, composed of two layers with 6 and 1 nodes respectively, combines the multiomics embedding with the drug features embedding and predicts the cell’s response to the drug, measured as the AUC. During the training phase, the input data were split into three sets: training (80%), testing (10%), and validation (10%) sets. Overall the network contains 12 734 neurons with at maximum seven layers. We have used Adam optimizer (initial learning rate of $10^{-5}$), for a total of 300 epoches.

### 2.2 Datasets

We used the Genomics of Drug Sensitivity in Cancer database (GDSC) (Yang *et al.* 2013) and the Cancer Therapeutics Response Portal v2 (CTRP) (Basu *et al.* 2013) to collect 383 998 triplets representing cell line, drug, and cell survival after treatment measure as AUC value. Overall, our dataset contains 889 cell lines and 684 drugs. Each drug is represented by 1009 variables, 881 molecular fragments from PubChem fingerprints, and 128 molecular descriptors from the software VolSurf+ (Crivori *et al.* 2000), as detailed in Supplementary Table S1. To represent the molecular properties of a cell line, we use the mutation and copy number profiles stored in three binary vectors, where the value corresponds to the presence or absence of a mutation/deletion/amplification in a particular gene in a given cell line, which were downloaded from the GDSC data portal (Yang *et al.* 2013). We selected 4870 (top 2.5%) frequently mutated genes in cancer using the pan-cancer compendium encompassing 33 cancer types and >10 000 tumor-normal exome pairs (Ellrott *et al.* 2018). Analogously, 2612 and 3625 genes contained in focal recurrently amplified copy number segments and deleted copy number segments respectively, selected as described in (Iorio *et al.* 2016). These genes were further filtered for those associated with at least one GO term present in the MOViDA hierarchy, obtaining 2931 and 2097 genes for amplifications and deletions, respectively. Gene expression was also used to compute a NES using single-sample gene set test using the Mann–Whitney-Wilcoxon Gene Set test (mww-GST) available in the yaGST package (Frattini *et al.* 2018). NES is an estimate of the probability that the expression of a gene in the geneset is greater than the expression of a gene outside this set: $\text{NES}=1-\frac{U}{mn}$, where *m* is the number of genes in a gene set, *n* is the number of those outside the gene set, U=mn+m(m+1)−T, and *T* is the sum of the ranks of the genes in the gene set.

For drug combination, we used the Therapeutic Target Database (TTD) (Zhou *et al.* 2022) to identify potential synergies among drug targets and then used the dataset of pharmaceutical synergies specific to breast, colon, and pancreatic cancer cells created by Jaaks *et al.* (2022) for validation.

To further validate our model’s capabilities, we extended its application to predict drug combination therapies utilizing the dataset presented by O’Neil *et al.* (2016). We selected the cell lines and drugs with available features, resulting in a dataset of 32 compounds and 32 cell lines, totaling 13 376 instances of combined cell line and drug treatments, with 1296 instances considered synergistic. To assess the synergistic interaction between drugs, we employed the Loewe Additivity score (Loewe 1953), utilizing a threshold of 30 to differentiate synergistic from non-synergistic outcomes.

### 2.3 Data imbalance strategies

Drug sensitivity data exhibits a significant skewness, characterized by many screens with low sensitivity outcomes (AUC close to 1) and very few with high sensitivity (AUC close to 0). To mitigate the potentially deleterious effects of this data imbalancing during the training, we used a weighted random sampler based on a multinomial distribution estimated from the data.

The AUC sensitivity scores are divided into twelve equally spaced bins between 0 and 1.2, and we used the inverse frequencies with additive smoothing to fix the weights of the multinomial sampler:


(2) $$f_{i}=\frac{c_{i}}{\sum_{j=0}^{c}c_{j}}v_{i}=\frac{1}{f_{i}}+\epsilon w_{i}=\frac{v_{i}}{\sum_{j=0}^{c}v_{j}},$$

where *c_i_* is the number of samples in bin *i*, *c* is the number of bins, *f_i_* is the relative frequency of the bin *i*, and *ϵ* is the smoothing penalty term.

We also used a *weighted loss* function to penalize errors associated with lower scores of ground truth and predictions. Hence we adopted the following *double-weighted MSE loss*.


(3) $$L(p,t)=\text{max}(w_{c_{p}},w_{c_{t}})*{\left(p-t\right)}^{2}.$$

Here, *p* is the prediction of a model, *t* is the ground truth, *c_p_* and *c_t_* are the corresponding bins and $w_{c_{p}}$ and $w_{c_{t}}$ are the weights associated with these bins as computed in Equation (2). This loss function guarantees higher weights for errors when either the ground truth or the prediction are in a class with few samples and, at the same time, lower weights for predictions when they are far from the ground truth.

We use an evaluation measure developed in the field of ordinal regression (Baccianella *et al.* 2009). This is motivated by the fact that discrete sensitivity levels can be considered ordinal variables, and the ordering between the values is significant, as they represent degrees of sensitivity. A simple and efficient approach to measure the performance in ordinal regression tasks for imbalanced datasets is the *macroaverage MSE* (MMSE) which is based on a sum of the classification errors across classes. where *c_i_* represents the set of samples in class *i*, *c* is the number of classes, *t_x_* id the ground truth of sample *x*, and *p_x_* is its prediction. The macroaverage MSE does not depend on the frequency of each class, as every class contributes to 1/c of the total measure. Therefore trivial assignments are penalized, whereas to have better *MSE^M^* the errors in all classes should be minimized.


(4) $$\text{MMSE}=\frac{1}{c}\sum_{i=0}^{c}\frac{1}{\left|c_{i}\right|}\sum_{x\in c_{i}}{\left(p_{x}-t_{x}\right)}^{2},$$

### 2.4 Model explanation

The Biology informed nature of MOViDA, as well as of DrugCell (Kuenzi *et al.* 2020), P-NET (Elmarakeby *et al.* 2021), and PASNet, (Hao *et al.* 2018) allows performing accurate post-hoc analyses. This enables us to identify the biological processes that contribute to the prediction of a cell line’s drug sensitivity the most. The state-of-the-art methodologies, such as LIME, DeepLIFT, DeepExplain, and SHAP (Kokhlikyan *et al.* 2020), are not suited to our case since most of them allow us to measure the contribution of either an individual input node or a full layer. Instead, our visible network is composed of sub-modules. Therefore, we developed an interpretation score, relative improvement score (*RIS*), specifically tailored for our model that measures the relative contribution of a submodule concerning its children in the GO hierarchy. We use *ablation* to quantify the importance of the VNN modules to the network output (Morcos *et al.* 2018).

First, we calculate the prediction for a specific drug-cell line pair. Then we recalculate the prediction after silencing the output of each submodule one by one, setting weights and biases to zero. Similarly, we silence all children subsystems for each GO term and obtain the third prediction. In the case of leaf nodes, we silence the corresponding inputs. The *RIS* score expresses the importance of a term during the prediction phase and its ability to combine the information from its children, comparing the deviations from the actual prediction of the models ablating first the father and then its children modules. The *RIS* is computed as follows: where *p* is the effective prediction for a specific drug-cell line pair, *p_f_* and $p_{c_{f}}$ is the prediction obtained by silencing a GO subsystem and its children, respectively. *RIS* is the interpretation score. For a given GO subsystem, positive RIS values correspond to a larger deviation in predictions when silencing children compared to the father.


(5) $$s_{f}=|p_{f}-p|\;\;\;\;\;\;\;\;\;\;s_{c_{f}}=|p_{c_{f}}-p|\;\;\;\;\;\;\;\;\;\;\text{RIS}=\frac{s_{c_{f}}-s_{f}}{s_{c_{f}}+s_{f}},$$

The advantages of *RIS* over the score adopted in DrugCell (RLIPP) are that: (i) it can be calculated for each individual drug-cell line pair and (ii) there are multiple ways to aggregate these values, by drug or by specific cell line types.

To further investigate the model, we have inspected all the elements that compose the inputs describing the drugs. The importance score of each feature was performed using DeepLift (Kokhlikyan *et al.* 2020).

### 2.5 Drug combination strategies through relevant subsystems

We used the RIS score calculated from a drug/cell-line pair to evaluate the potential effect of drug combination on the dataset described in (Jaaks *et al.* 2022). We selected the top five enriched GO terms along with associated genes. From the collection of drug targets *TTD* (Zhou *et al.* 2022), we obtain the drugs targeting the genes associated with the previous selection of GO terms, marking them as potentially synergistic for that drug-cell line pair. We then compared our predictions on the synergistic dataset, marking the right (TP) and wrong (FP) combinations and comparing the ratio of TP to FP and the ratio of synergistic drugs to non-synergistic drugs to understand if the former was significantly higher than the latter. We took all drug combinations studied for a specific cell line and drug and counted how many of these combinations were synergistic (S) and how many are not (NS). We applied the binomial test on TP over (TP + FP), which is the Precision metric, with probability equal to S/(NS+S), thus accounting for the number of synergistic combinations. The *P*-values for the binomial test and the enrichment scores $\frac{\text{TP}/(\text{TP}+\text{FP})}{S/(\text{NS}+S)}$ of the above-described tests are used in the volcano plots reported in Section 3.

### 2.6 Extension for drug synergy prediction

We have also evaluated our model in predicting synergistic effects of drug combinations as well. For this purpose, as in Siamese neural networks, we replicated the right branch of our model. The final ANN concatenates the cell line and drugs embedding. In this way, the order of drugs is important, so we doubled the initial dataset by considering the two possible combinations of drug pairs. In this case, the model had 16 hidden nodes for each GO submodule. Also, the model was trained for a total of 50 epochs, since it converged faster given the smaller amount of samples, and penalty term *ϵ* was set to five, as the weights were used only for weighted random sampling. Prediction of synergy was posed as a binary classification problem, distinguishing the cases where there is synergy or not. Given the presence of imbalance in the dataset, we used focal loss (Lin *et al.* 2017) with hyperparameters *α* and *γ* set to 0.4 and 2, respectively.

## 3 Results

### 3.1 Dataset imbalance

MOViDA was trained to predict the response of a cellular model to a specific drug, measured as AUC. AUC combines information about the potency and efficacy of the drug into a single measure (Fallahi-Sichani *et al.* 2013). A value close to zero means high sensitivity, a value close to 1 represents no effect of the drug, if >1, the drug has the effect of promoting cell viability. Besides the high interest in accurate predictions for drugs with high sensitivity, the majority of drug screens typically have AUC values that are close to 1, therefore the distribution of the AUC is particularly skewed. Supplementary Fig. S1a shows the distribution of the AUC values after binning AUC values into 12 bins (10 bins for the interval between 0 and 1 and other two for values >1): class 0 (AUC scores in the range [0.0, 0.1]) was 80 times less populated than class 9 (scores in the range [0.9, 1.0]). To mitigate this effect, our approach considered a weighted random sampler and a double-weighted loss (section). Both use the weights calculated as a function of the inverse frequencies of each class plus a smoothing term *ϵ*. Supplementary Fig. S1b shows the number of samples for all classes: besides the raw case (no weights), different scenarios are depicted by varying the *ϵ* parameter that affects the weights. The ideal scenario lies between the raw case and the perfectly balanced dataset (with *ϵ* set to 0), which, on the contrary, could produce too many sample repetitions. After parameter tuning, we choose the value of *ϵ* equal to 80 as a good compromise, producing, on average, a 4-fold repetition for the samples in a less represented class. The improvement of these strategies is shown Supplementary Fig. S2. The weighted random sampler had a greater impact than the double-weighted loss on the results. However, the combination of both approaches not only reduced the error but also enhanced robustness with a much lower variability across validation folds.

### 3.2 Performance and comparison with DrugCell

**Figure 2.**
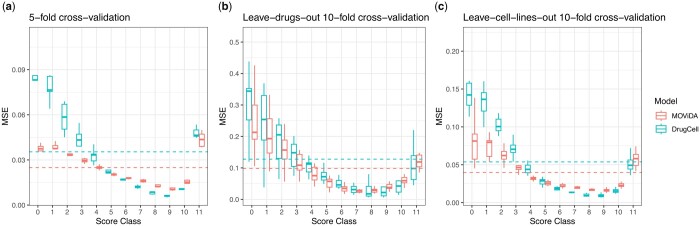
Evaluation and comparison. (a) Five-fold cross-validation results for each model’s mean squared error (MSE) per class. The plot illustrates the lowest, mean, and highest MSE values obtained. The dashed lines correspond to the macroaverage MSE averaged over cross-validation. (b and c) Ten-fold cross-validation in which each fold comprised a unique set of 10% cell lines or drugs that were not included in the other folds.

The accuracy of the prediction was evaluated by measuring the Spearman and Pearson correlation between the predicted AUC values and the actual ones, averaged over 5-fold cross-validation. In order to consider the imbalance, the correlation was computed by sampling an equal number (*n* = 100) of examples from each class, and this process was repeated for 1000 runs. The results show that both Pearson and Spearman correlation were 0.89. The DrugCell model showed good results as well, with Pearson correlation of 0.86 and Spearman correlation of 0.88 (Supplementary Table S2). When we used the macroaverage MSE (MMSE), which accounts for the imbalance of the classes, MOViDA had a lower error (0.025 vs. 0.035) after the cross-validation. Indeed MOViDA can make much more accurate predictions of the AUC in classes with fewer training examples (classes between 0 and 5). Those classes are the most meaningful ones as they represent cases of high sensitivity to drugs. Notably, MOViDA exhibited higher accuracy in these classes compared to DrugCell, with a significantly lower error rate (0.032 vs. 0.060). Conversely, for classes with a larger number of training examples (classes 6–11), MOViDA and DrugCell perform similarly, with comparable error rates (0.020 vs. 0.018). We show in Fig. 2a, the MSE calculated for each class which depicts a specific trend. MOViDA performs slightly worse for the upper classes (6–11) than DrugCell, but significantly better for the lower classes (0–5). To better visualize this behavior, if we cast the regression the sensitivity values as a classification problem in terms of prediction of AUC interval classes, the confusion matrices in Supplementary Fig. S3a and b show that DrugCell tends to over-estimate the majority class. In contrast, MOViDA has better accuracy along the cells on the diagonal.

We investigated the robustness of each model using two cross-validation strategies: leave-cell-lines-out and leave-drugs-out, both implemented in a 10-fold nested scheme. We created 10 folds for each strategy, ensuring that each fold contained cell lines or drugs not present in the other nine folds. These cross-validation strategies enabled us to assess the ability of the models to generalize to unseen data (cell lines of drugs) and evaluate their accuracy. As reported in Supplementary Table S2 and Fig. 2b and c, while all models experienced a slight drop in accuracy during leave-cell-lines-out cross-validation, MOViDA consistently outperformed DrugCell (0.040 vs. 0.054). In contrast, during leave-drugs-out cross-validation, MOViDA remained stable across the 10 folds, while DrugCell exhibited higher variability.

The performance of MOViDA was further evaluated through comparative analyses, varying the type of drug representation used as input: Morgan Fingerprint, PubChem Fingerprint, and VolSurf+ descriptors (Supplementary Fig. S4a). Results indicated that MOViDA exhibited the lowest MMSE compared to other models, particularly with smaller errors in the lower classes. Furthermore, we have tested MOViDA by replacing the ANN for drug embedding with another one that contained more than 5-fold the number of parameters, with 512, 128, 32, and 8, nodes respectively, and four linear layers (Supplementary Fig. S4b). The results showed that increasing the size of the ANN did not improve performance. Finally, MOViDA was compared with a Multi-Layer Perceptron (MLP) consisting of five linear layers (1024, 256, 64, 4, and 1 nodes each) with ReLU activation functions to assess whether the trade-off between explainability and performance exists. The results indicated that MOViDA and the standard network performed similarly, with comparable MMSE.

### 3.3 The RIS score identifies pathway dependencies in specific cellular models

We implemented the RIS based on ablation of modules representing GO terms in the VNN. This score can calculated for each specific cell line-drug prediction, so we can show which GOs are most predictive for a specific case or tissue (represented as a group of cells) or drug.

**Figure 3.**
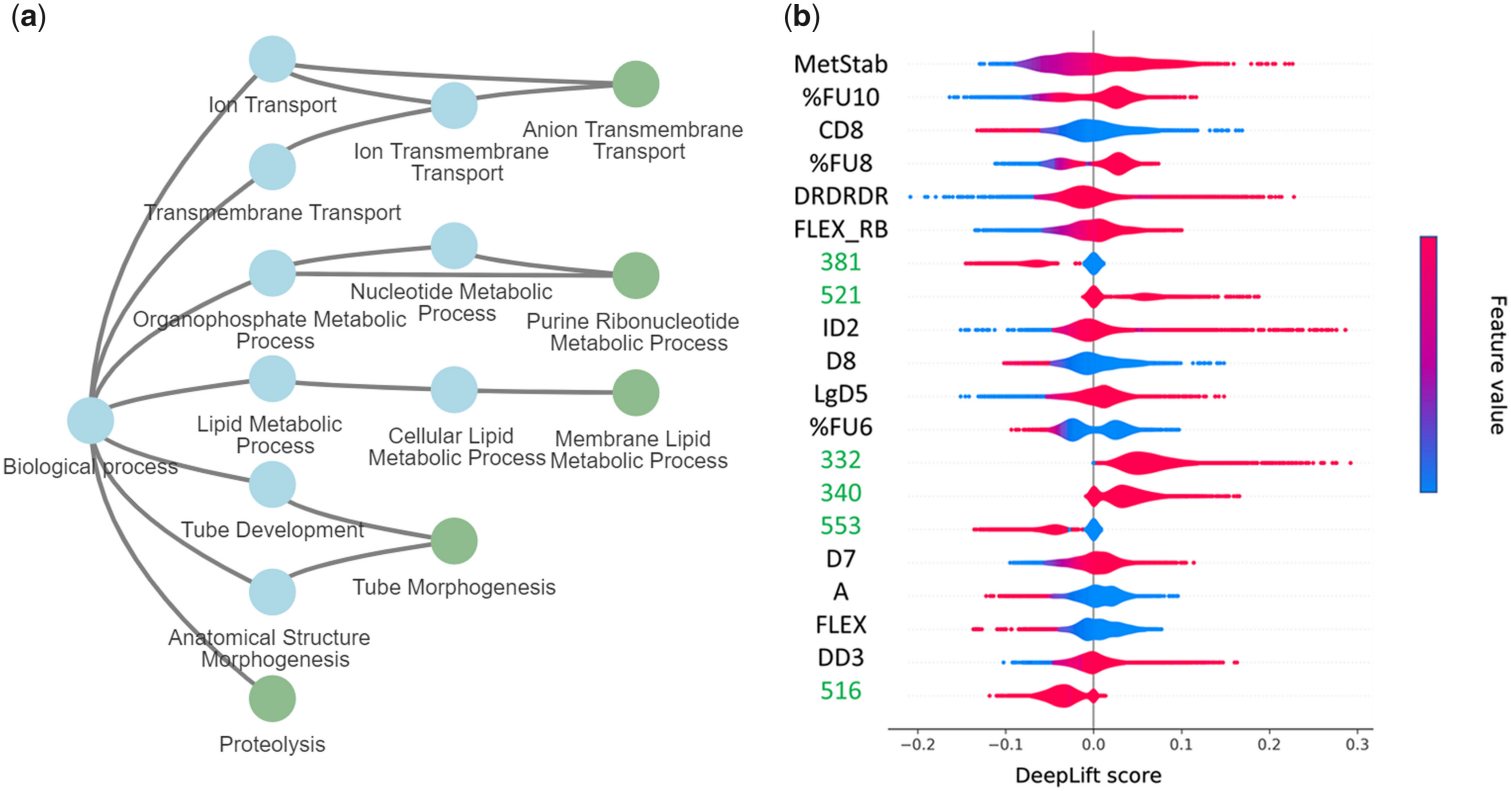
Explainability. (a) Top 5 RIS score associated to GOs (leaf nodes), considering the ALLSIL cell line and GSK1070916 drug. The whole subtree is displayed. (b) Deep lift drug feature interpretation of Liver tissue. If feature values are low on the left side of the violin, AUC is directly dependent on the feature. Many VolSurf+ descriptors emerge as the most important features, respect to PubChem fingerprint features represented in the figure as bit positions.

Among the leukemia cell lines, we selected the ALLSIL cell line highly sensitive to GSK1070916, an ATP-competitive inhibitor of Aurora kinase, which is important during cell division. The RIS scores associated with this prediction revealed that *anion transmembrane transport* (GO:0098656) was among the most important modules for prediction (Fig. 3a). The overexpression of ATP-binding cassette (ABC) transporters, particularly ABCG2, contributes to reduced cytotoxicity of GSK1070916 (Wu *et al.* 2021). The family of these genes is responsible for transporting substances across the cell membrane using the energy produced by ATP electrolysis. Interestingly, ALLSIL is ABCC9 mutant which, together with ABCG2, is downregulated in this cell line. Similarly, *proteolysis* (GO:0006508) had a high RIS score for this cell line-drug pair. This can be attributed to AURKB (aurora kinase B) phosphorylating caspase-2 by mediating its proteolysis (Lim *et al.* 2021). As a result, cell division is not stopped. In our case, GSK1070916 inhibits AURKB promoting apoptosis of the cancer cell. AURKB is over-expressed in the ALLSIL cell line.

A high RIS score was also reported for the *positive regulation of the reactive oxygen species (ROS) metabolic process* pathway (GO:2000379) associated with the DB cell line (lymphoma) when administered with Dinaciclib (an inhibitor of CDK1, CDK2, CDK5, and CDK9). Interestingly, it has been recently reported that the inhibition of CKD leads to increased mitochondrial ROS levels, confirming this pathway’s importance in the cellular response to this exposure (Riess *et al.* 2021).

Another interesting case is the response of the cell line 5637 (urinary bladder) to Thapsigargin. This molecule operates by targeting the calcium pump, which leads to an increase in calcium concentration within the cell. RIS score selected several GO terms (Supplementary Fig. S5) associated with “Calcium Ion Transmembrane Transport” (GO:0070588). Additionally, thapsigargin induces the production of interleukin-2 (IL-2), which in turn stimulates the growth of T cells. This effect is captured by MOViDA that gives a high RIS score to the term “Positive Regulation of T Cell Proliferation” (GO:0042102) (Kim *et al.* 2018). Furthermore, several studies suggest that tyrosine kinase activity plays a role in thapsigargin-induced calcium influx (Lee *et al.* 1993).

### 3.4 Drug features interpretability

As a complementary interpretation step, we can also measure the impact of individual drug features on the model’s predictions with the DeepLift score (Shrikumar *et al.* 2017). Figure 3b shows the 20 most important features of our model. The importance lies in the variability of the score for the various cell lines: the more it varies, the more significant it is for predictions. The most relevant feature was the VolSurf+ descriptor METSTAB for all the cell lines. Such descriptor refers to metabolic stability (measured on human liver microsomes), mostly due to isoform 3A4 of the cytochrome P450 system. We noticed a direct relationship between such a feature with the AUC. This means low values for metabolic stability (thus, fast CYP3A4-mediated metabolism) for high-sensitivity drugs. This agrees with the absorption, distribution, metabolism, and excretion profile of many anticancer drugs, most of which are metabolized in the liver by CYP3A4. Several features were related to drug lipophilicity; among these, the VolSurf+ descriptors D8 and CD8 are associated with highly lipophilic regions of the molecules, and characteristics of active molecules (low AUC values).

Two features measure the molecular flexibility, namely the VolSurf+ descriptors FLEX and FLEX_RB. Given their lift values, we can argue that for most of the predictions, flexibility is inversely related to AUC, whereas the number of rotatable bonds is directly related to AUC. Although it is uncommon to have an opposite behavior for these two features, an attempt to generalization may be that anticancer drugs are generally flexible but with a low number of rotatable bonds (compared to the overall number of bonds). The VolSurf+ descriptors %FU8 and %FU10 can measure the percent of the unionized fraction at a given pH (8 or 10). According to violin colors, the system identified a direct relationship with AUC; in other words, many anticancer drugs have strong or weak acid groups that are reflected onto the significant presence of ionized species at basic pH.

### 3.5 Drug combination predictions

**Figure 4.**
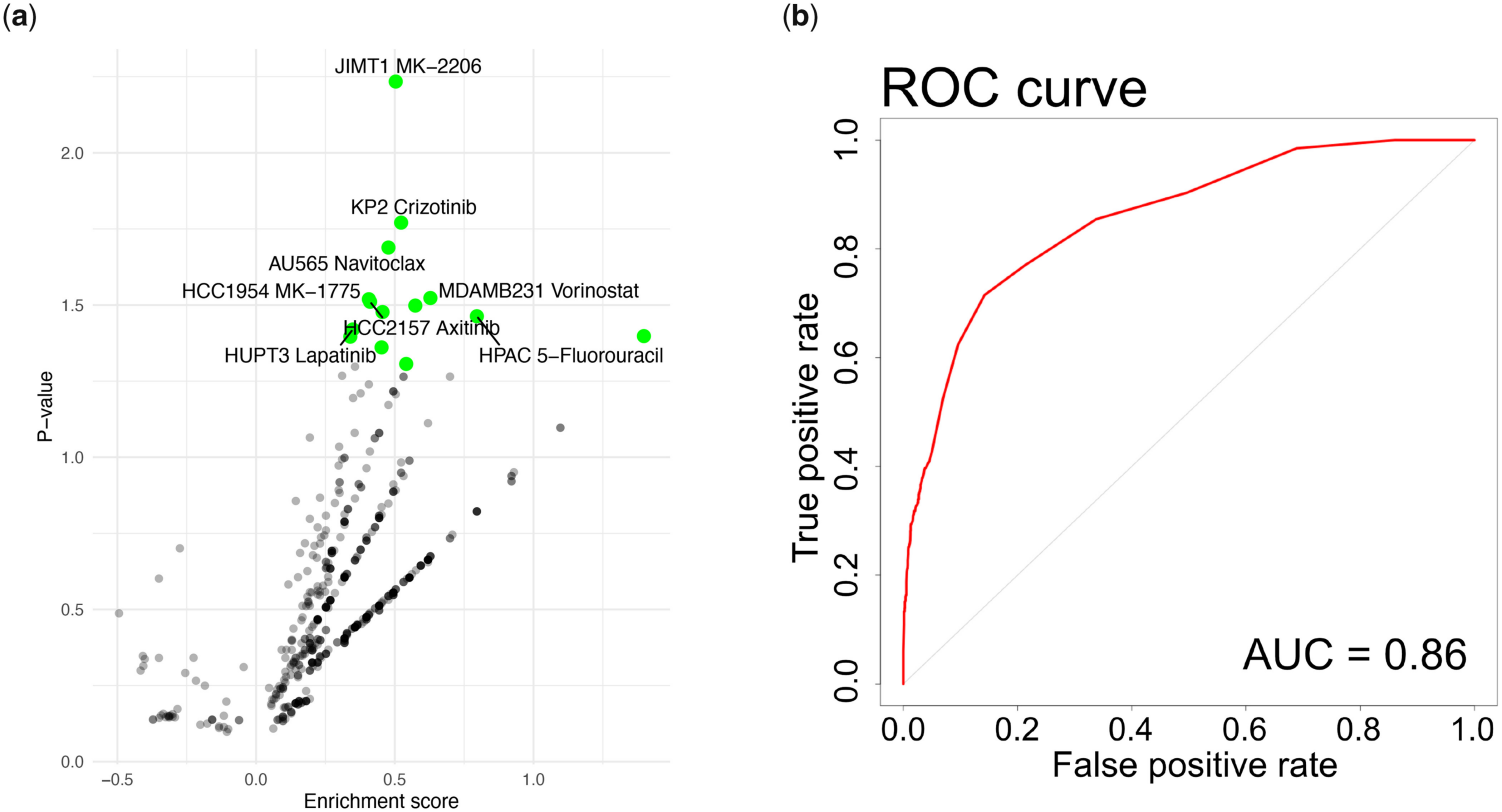
Evaluation of drug synergy prediction. (a) Enrichment scores against the *P*-values of binomial, testing the Precision of the model, using as the probability the percentage of synergistic combination. The green points correspond to drug-cell lines pairs that have a significant number of positive drug candidates, considering the numerosity of synergies in the dataset. (b) ROC curve for predicting the synergistic effect on the dataset O’Neal (O’Neil *et al.* 2016) with 32 compounds and 32 cell lines, totaling 13 376 instances of combined cell line and drug treatments, with 1296 instances considered synergistic.

MOViDA predictions can be used to uncover potential drug synergies. Given the interpretation score for specific drug-cell line pairs, we selected the genes involved in GO terms with the highest scores and prioritize as potential combinations the drugs targeting these genes. The volcano plot in Fig. 4a shows the cell line-drug pairs for which the candidate molecules are enriched for experimentally validated synergistic drugs. For example, the synergy predictions associated with the breast cancer cell line JIMT1 (breast ductal adenocarcinoma) and the drug MK-2206, a highly selective inhibitor of Akt1/2/3, has among the top 5 scoring GO categories the GO:0007169 (transmembrane receptor protein tyrosine kinase signaling pathway) and the GO:0007584 (response to nutrient). Our model selected Lapatinib, PD173074, Axitinib, Linsitinib, Sapitinib, and OSI-027 as potential candidates for combination therapy with MK-2206. They are all tyrosine kinase inhibitors involved in tumor cell growth. The association between these drugs and MK-2206 is well documented in the literature, as many tyrosine kinases are part of the PI3 kinase-AKT cascade, affecting mTOR activity (Lara *et al.* 2015). Another relevant combination was Navitoclax and Vorinostat associated with the MDAMB231 cell line (triple-negative breast cancer). The latter is an HDAC inhibitor, which decreases the expression of BCL2 family proteins (Duan *et al.* 2005). Since Navitoclax is an inhibitor of this anti-apoptotic protein family, it has been shown that its efficacy, combined with Vorinostat, can induce apoptosis in cancer cells (Nakajima *et al.* 2016).

We also extended our model to classify drug synergy. MOViDA was able to achieve an AUROC of 0.86 (Fig. 4b) and AUPR of 0.55 (Supplementary Fig. S6), which indicates high accuracy in classifying drug synergy despite the imbalanced nature and small size of our dataset. We also performed a comparative analysis between our model and a shallow neural network consisting of four linear layers with batch normalization, trained using similar hyperparameters to MOViDA. Our model yielded superior performance, with both areas exceeding those of the compared model (Supplementary Fig. S7).

## 4 Conclusion

In this article, we presented MOViDA, a biologically informed neural network architecture for the prediction of drug sensitivity of cellular models of cancer. The assessment of anti-cancer drugs and the identification of potential synergistic effects can be ideally assessed by using patient-derived cell lines (Liu *et al.* 2016). However, this process requires substantial time, and there is no guarantee of efficiency. The use of ML to exploit the variety of screening data already available, together with the knowledge of the molecular features of cellular models, can help to accelerate the process of drug prioritization for experimental validation (Dezső and Ceccarelli 2020) and candidate combination therapies (Jaaks *et al.* 2022). The adoption of a biologically informed architecture has three main advantages: (i) it allows to uncover the role of specific pathways in response to drug stimuli; (ii) it improves the trust in predictions, especially among non-ML experts; and (iii) the efficient parameterization of our model can simplify the learning process rather than use arbitrarily overparameterized, architectures for prediction, simplifying interpretability. Most drug sensitivity prediction models only use gene expression data (Chen *et al.* 2021), however, the effect of single nucleotide mutations, DNA methylation and DNA copy number variation on drug sensitivity should also be considered. Here we have presented a visible neural with an improved accuracy level due to the use of multiple omics platforms and the better handling of imbalance of data. We also have developed an interpretability score that has the advantage of producing a value for every cell line-drug pair and, therefore, can be summarized in terms of cellular models derived from the same tissue/cancer subtype or at the level of individual drugs. We have shown that our score produces meaningful results that can be the subject of experimental follow-up. We have also introduced a set of features that can be directly related to behavior or chemical groups. We confirmed the importance of chemical features such as LogP, FLEX as well as %FU(4–10) also observed in the inhibition of glycoprotein (Broccatelli *et al.* 2011). In conclusion, the model has been successfully extended for drug synergy prediction while maintaining its interpretable nature.

## Supplementary Material

btad432_Supplementary_Data
