# Supplementary material for: MOViDA: multiomics visible drug activity prediction with a biologically informed neural network model
Source: Bioinformatics. 2023 Jul 11;39(7):btad432. doi: 10.1093/bioinformatics/btad432 (PMC10375315; doi:10.1093/bioinformatics/btad432)
Supplement: btad432_Supplementary_Data [file btad432_supplementary_data.zip › BIOINF-2023-0608R1_SuppelmentaryInfo.pdf]

Table S1. Illustration of VolSurf+ descriptors

VolSurf+ descriptors	Description
Size and shape descriptors	Molecular volume, surface, rugosity, globularity (how much the molecule is spheroidal), flexibility parameters
Hydrophilic regions descriptors	Molecular hydrophilic volumes, Capacity factors (ratio of hydrophilic surface over the total molecular surface)
Hydrophobic regions descriptors	Molecular hydrophobic volumes, Capacity factors (ratio of hydrophobic surface over the total molecular surface), the difference between the maximum conformational hydrophobic volumes and the hydrophobic volumes
INTEraction enerGY (INTEGY) moments	Imbalance between the center of mass of a molecule and the barycentre of its hydrophilic or hydrophobic regions
Descriptors of H-bond donor/acceptor regions	The molecular envelope generating attractive H-donor or H-bond acceptor interactions
Mixed descriptors	Hydrophilic-Lipophilic balance (ratio between hydrophilic and hydrophobic regions), Amphiphilic moment, Critical packing parameter (ratio between the hydrophilic and lipophilic part of a molecule), average molecular polarizability,, dispersion of chemical in water fluid, Molecular Weight, Log P 1octanol/water, Log P cyclohexane/water, Log D, Polar and Hydrophobic Surface Areas
Charge State descriptors	Number of Charged Centers, Available Uncharged Species, % unionised species
3D pharmacophoric descriptors (TOPP)	Dry, H-bond donor, H-bond acceptor and mixed Dry, H-bond donor and acceptor 3D triplets pharmacophoric areas
ADME model descriptors	Intrinsic solubility, Solubility at various pH, Solubility profiling coefficients (distinguish compounds that present similar solubility but different pH-depended profile or vice-versa), CACO2 permeability, Skin permeability, % of protein binding, Volume of Distribution, High Throughput Screening Flag

Table S2. Comparison between MOViDA and DrugCell using: macroaverage MSE (MMSE) over all classes, lower classes (0-5), and higher classes (6-11), sampled Pearson correlation and sampled Spearman correlation. Each are average over k-fold cross-validation.

	MMSE	MMSE (0-5)	MMSE (6-11)	Pearson	Spearman
MOViDA	0.025	0.032	0.020	0.89	0.89
DrugCell	0.035	0.060	0.018	0.86	0.88
MOViDA leave-cell-lines-out	0.040	0.059	0.026	0.82	0.83
DrugCell leave-cell-lines-out	0.054	0.100	0.021	0.81	0.82
MOViDA leave-drugs-out	0.098	0.160	0.055	0.51	0.52
DrugCell leave-drugs-out	0.128	0.195	0.080	0.40	0.50

(a)

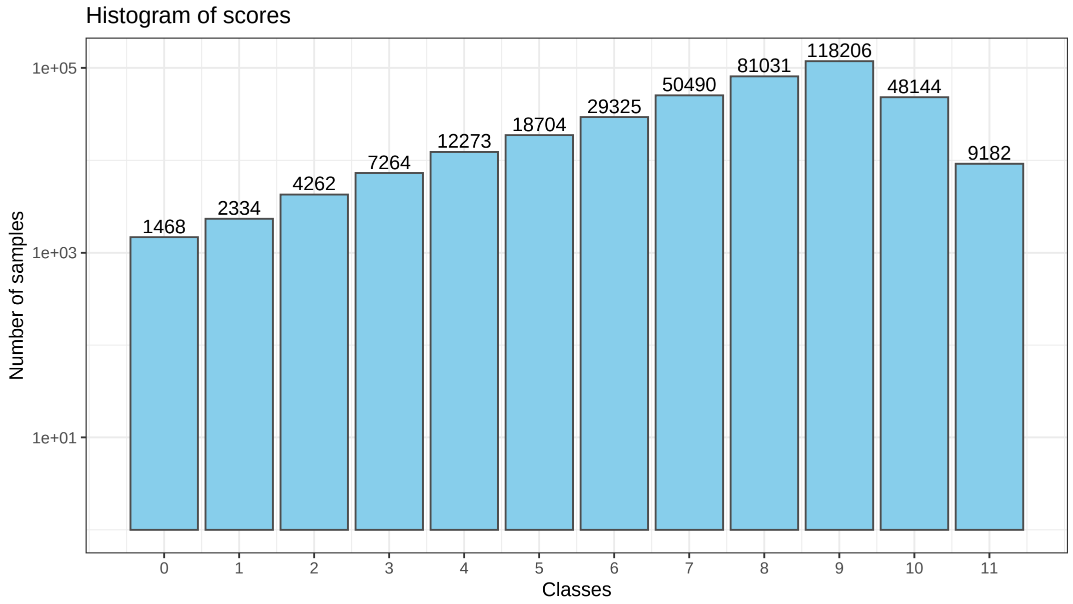

(b)

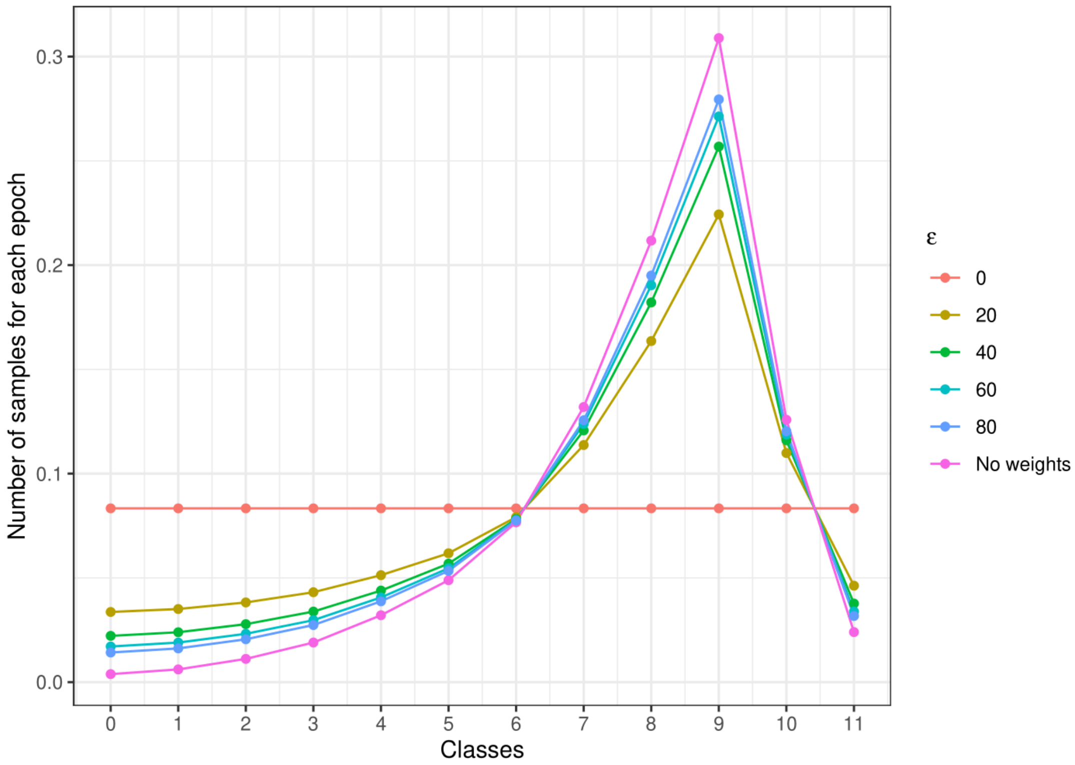

Figure S1: **Data Imbalancing review**. a) BarPlot distribution of AUCs divided in 12 classes, showing high data skewness, with very few examples for classes corresponding to high drug sensitivity (low AUC values). b) Probability to pick up a sample of a specific class by varying ϵ parameter. It is computed as $w_i * s_i$ normalized.

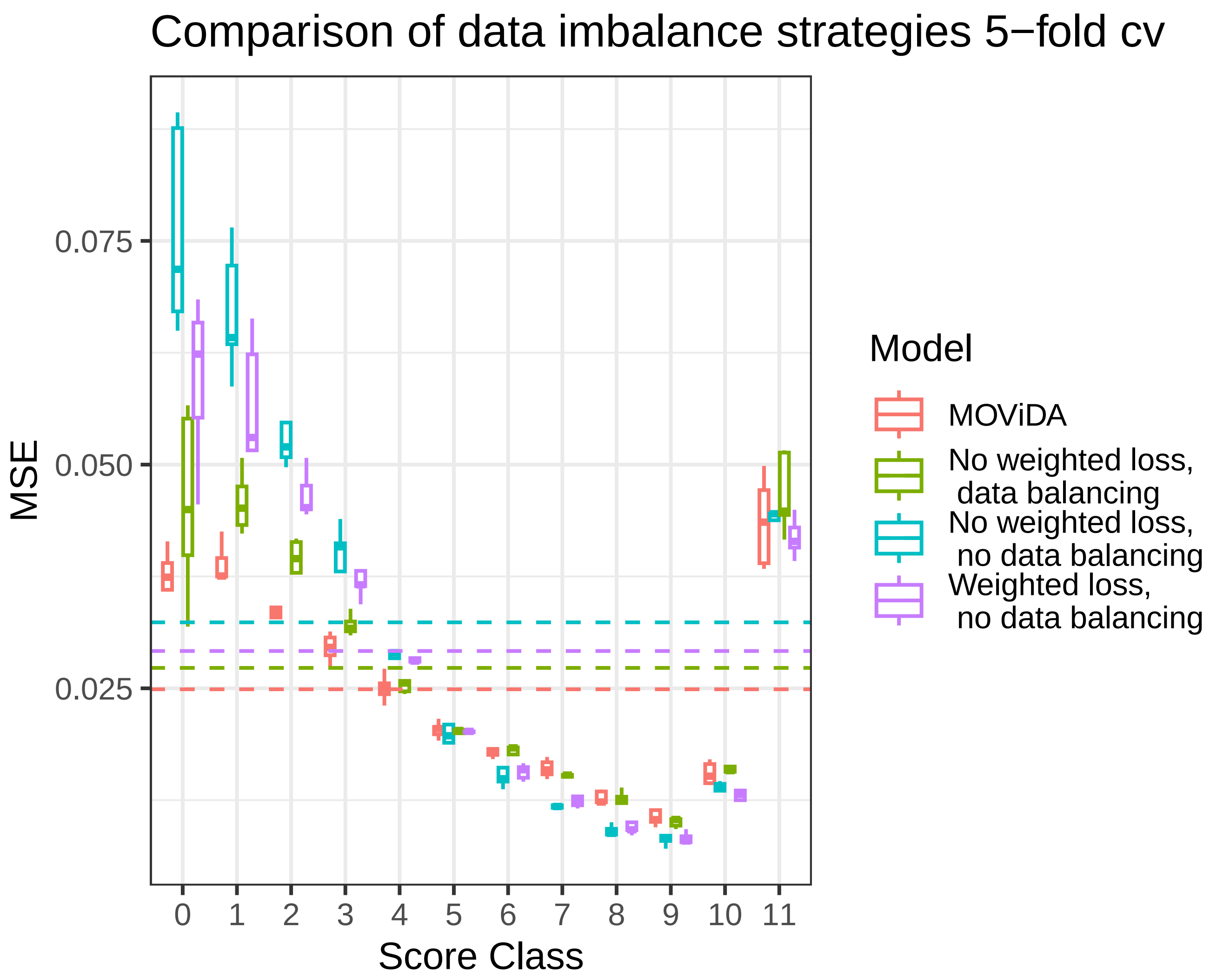

Figure S2: **Evaluation and comparison** MSE computed for each class with dashed line correspondind to the macroaverage MSE. Assessment of the impact of data imbalance and weighted loss on 5-fold cross-validation.

(a)

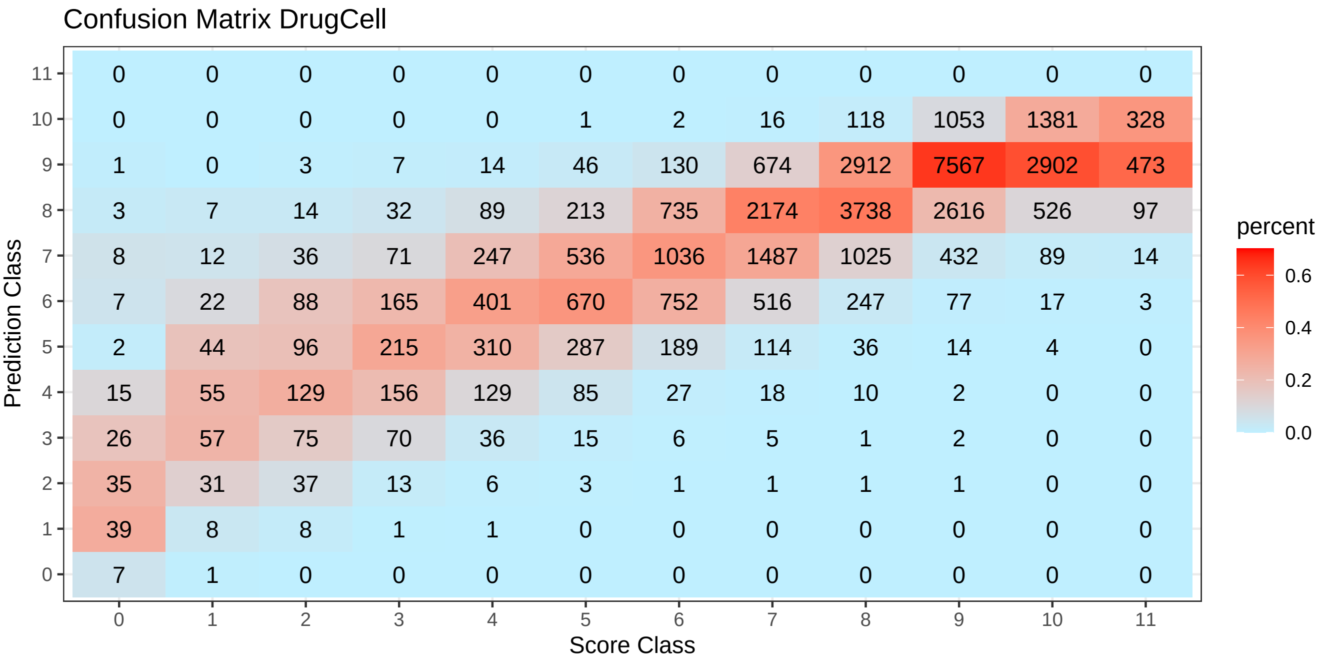

(b)

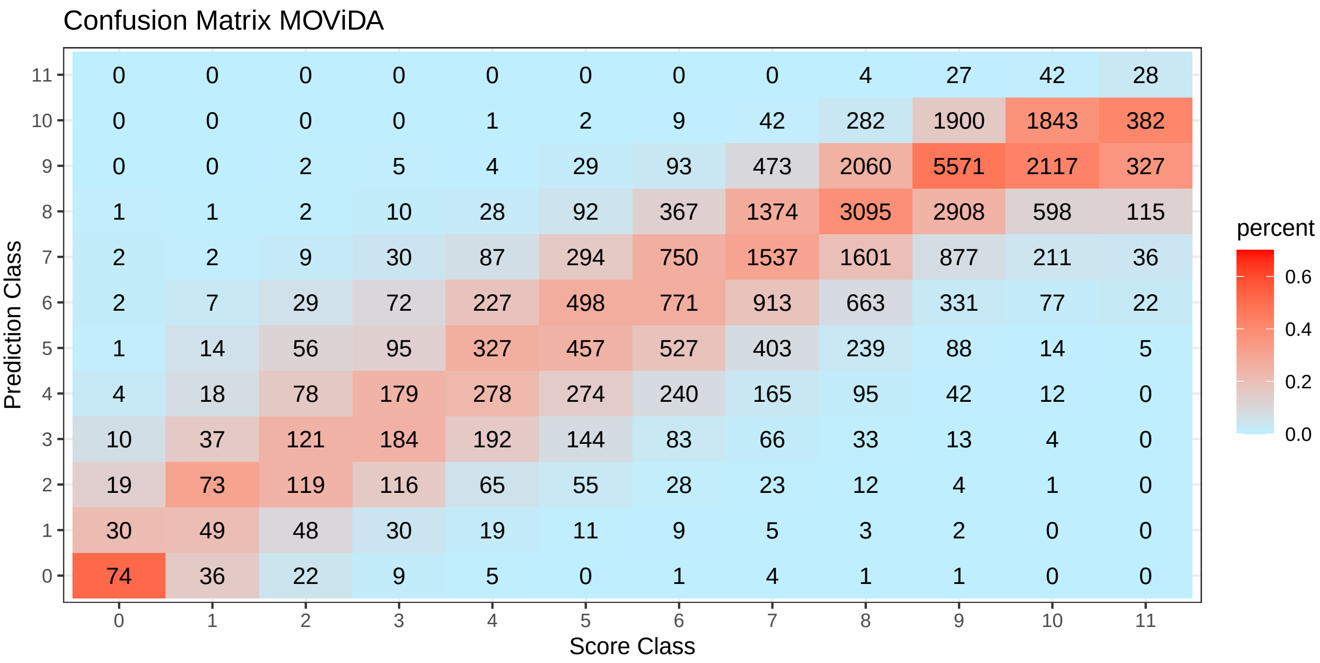

Figure S3: **Evaluation and comparison** Confusion matrices of DrugCell and MOViDA respectively, the percentage of each box is calculated based on the numerosity of the reference score class

(a)

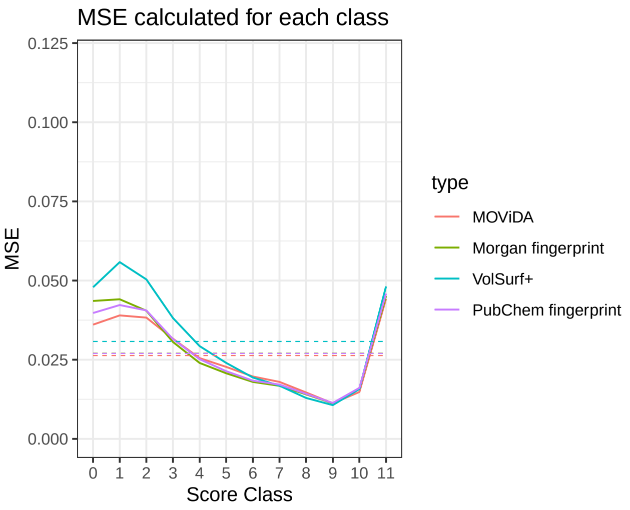

(b)

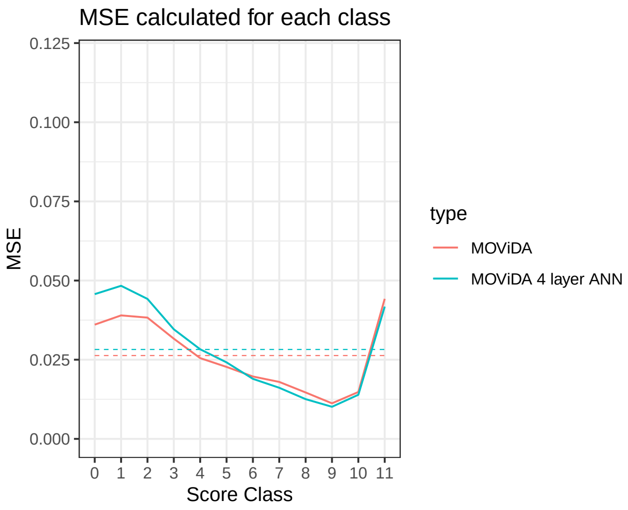

(c)

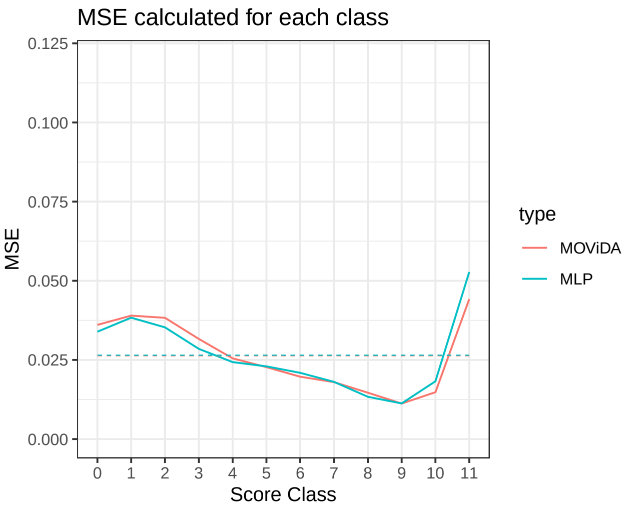

Figure S4: **Evaluation and comparison** MSE computed for each class with dashed line correspondind to the macroaverage MSE. Comparison of MOViDA: a) varying drug input (Morgan fingerprint, VolSurf+, PubChem fingerprint; b) substituting the ANN for drug embedding with 4 linear layers ANN of 512, 128, 32, 8 nodes respectively; c) comparison with a MLP, composed of 5 linear layers, with 1024, 256, 64, 4, 1 nodes respectively, and ReLU as activation functions.

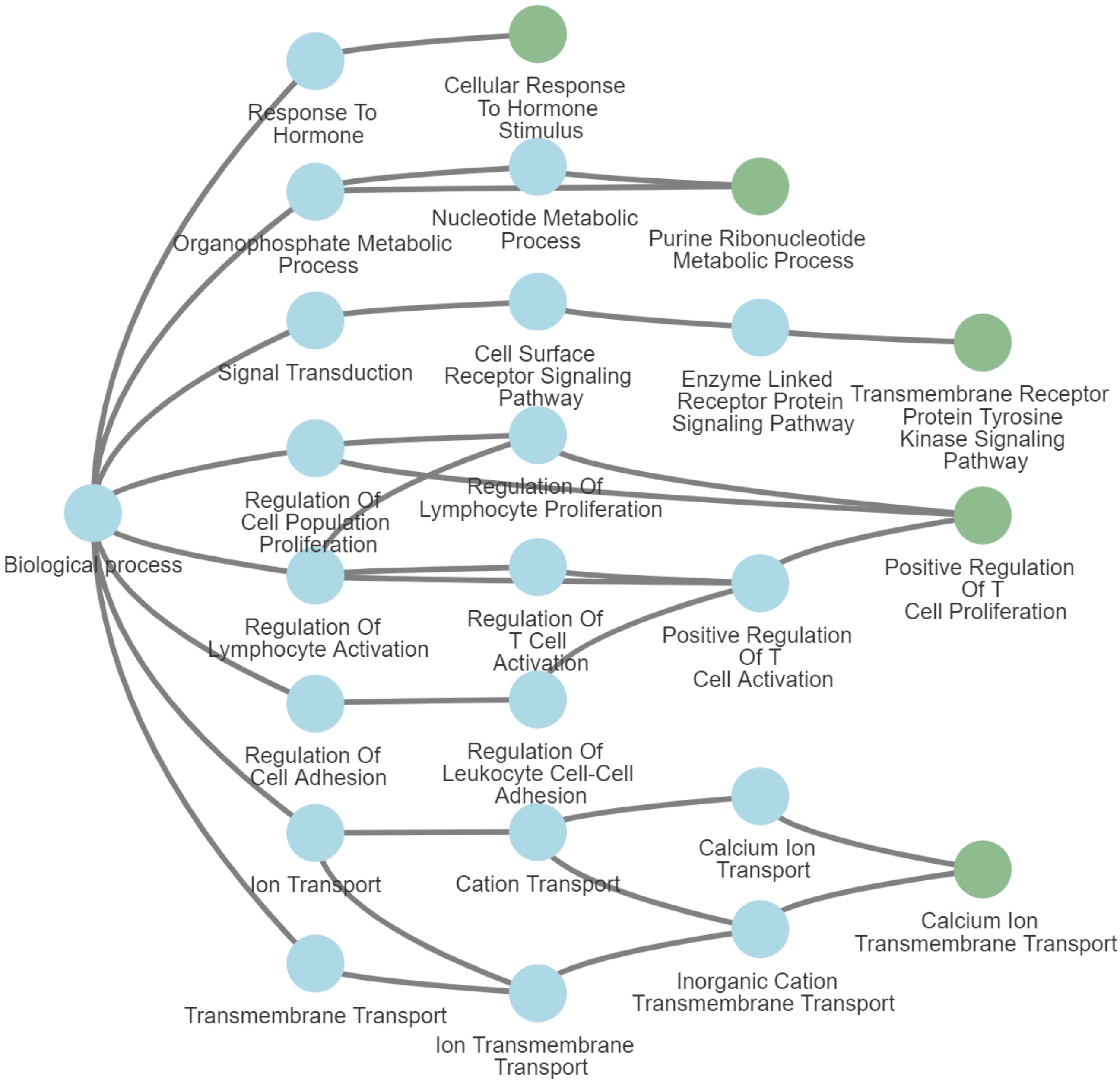

Figure S5: **Model identifies crucial GOs for drug sensitivity** Top 5 RIS scores associated with GOs (green nodes) in the displayed sub-tree (blue nodes), considering the 5637 cell line's sensitivity to the thapsigargin drug.

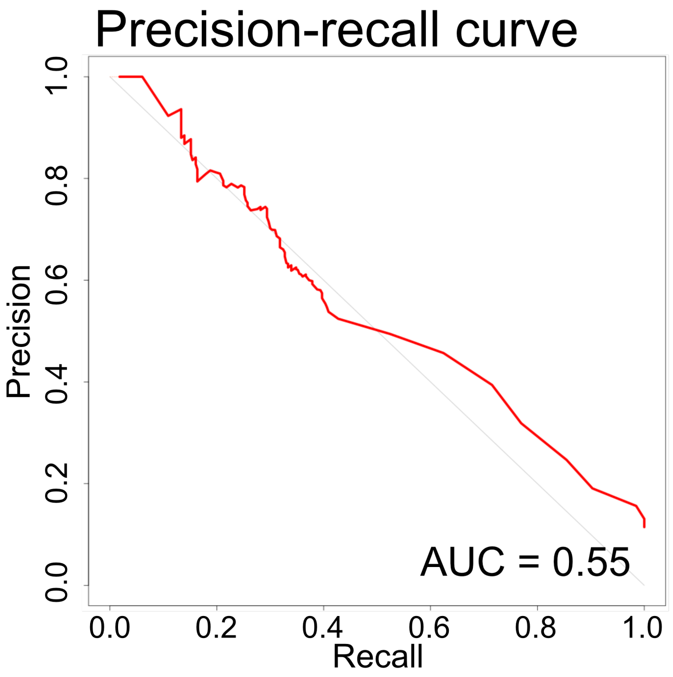

Figure S6: **Synergy prediction** Evaluation of synergy prediction performance using PR curves.

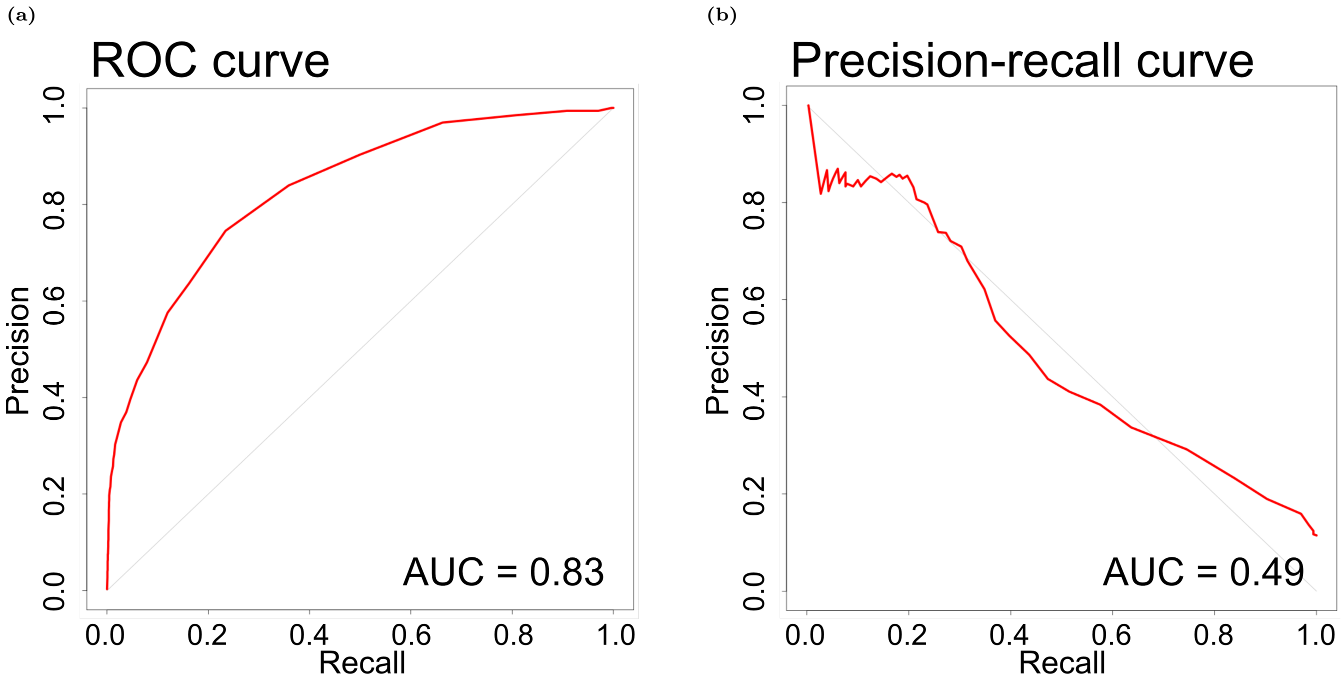

Figure S7: **Synergy prediction using MLP** Evaluation of synergy prediction performance using ROC and PR curves for a MLP.
